# Borderline personality traits in early adolescence: the role of attachment and reflective functioning

**DOI:** 10.3389/frcha.2026.1898910

**Published:** 2026-07-24

**Authors:** Iouliani Koullourou, Christos Mantas, Thomas Hyphantis, Evangelia Karagiannopoulou, Carla Sharp, Giovanni Abrahão Salum, Konstantinos Kotsis

**Affiliations:** 1 Department of Psychiatry, Faculty of Medicine, University of Ioannina, Ioannina, Greece; 2 Department of Psychology, University of Ioannina, Ioannina, Greece; 3 Research Department of Clinical, Educational and Health Psychology, University College London, London, United Kingdom; 4 Department of Psychology, University of Houston, Houston, TX, United States; 5 Child Mind Institute, New York, NY, United States

**Keywords:** attachment, borderline personality traits, early adolescence, outpatients, reflective functioning

## Abstract

**Background/objective:**

Borderline personality disorder (BPD) is a severe mental health condition typically identified in adolescence. Insecure attachment has been linked to BPD, with adult studies suggesting that reflective functioning might represent a mechanism linking attachment patterns to BPD. However, this interplay remains insufficiently explored in early adolescence—a developmental phase associated with reorganization of attachment and social cognitive function. The aim of this study is to explore the association between attachment styles and borderline personality traits (BPTs) in early adolescence and to test whether reflective functioning explains the association between attachment and borderline traits.

**Methods:**

A total of 112 adolescents aged 11–14 years attending a community outpatient child and adolescent mental health service completed the Attachment Questionnaire for Children (AQC), the Reflective Functioning Questionnaire (RFQ) and the Greek modified Borderline Personality Features Scale – Children (BPFS-C). Mediation analysis was conducted using secure attachment as the reference category.

**Results:**

Insecure attachment styles were significantly associated with higher odds of elevated BPTs. Reflective functioning significantly explained a significant amount of variance in the association between ambivalent attachment and high BPTs [OR = 2.92, 95% BCa CI (1.14, 10.17)], whereas avoidant attachment demonstrated a smaller but marginally statistically significant indirect effect through reflective functioning [OR = 1.77, 95% BCa CI (1.00, 3.95)] alongside a strong direct effect [OR = 4.34, 95% BCa CI (1.36, 12.53)].

**Conclusions:**

Avoidant and ambivalent attachment appear to be strongly linked to BPTs in early adolescence through partially distinct pathways, highlighting the need for early targeted interventions.

## 1 Introduction

Borderline personality disorder (BPD) is a severe mental health condition characterized by identity problems, instability of interpersonal relationships and emotion dysregulation (1). BPD affect 2%–3% of adolescents in the community, 11%–22% of adolescents attending outpatient mental health services, and up to 33% in inpatient adolescent services (1). As these rates were derived from different studies, they may not generalize uniformly across countries. As adolescence represents a developmental window that is particularly critical for expansion of the social brain (2), as well as the emergence of personality pathology, vulnerabilities in attachment security (characterized by avoidant or ambivalent patterns of relating) and impairments in reflective functioning (defined as the capacity to understand one's own and others' behavior in terms of underlying mental states) can be associated with core features of BPD (3). Identifying specific pathways in this age group may inform early detection strategies and guide preventive or early interventions.

Evidence from studies in adults, suggests that insecure attachment is associated with BPD, while a recent study in adult women identified mentalizing as a key mechanism linking insecure attachment to Borderline Personality Traits (BPTs) (4, 5). Recent evidence also suggests that BPTs typically become observable in adolescence, and elevated BPTs in adolescence show meaningful continuity and are associated with an increased likelihood of borderline pathology in adulthood (6, 7). Most existing studies focus either on attachment or on reflective functioning, or they examine adult populations where personality organization is more crystalized compared to adolescence (8).

Theoretical and conceptual models for the development of personality pathology propose that personality disorder develops against the backdrop of an insecure early caregiving environment, which in turn, impedes social-cognitive development (9, 10). Specifically, secure attachment relationships are thought to facilitate the development of mentalizing (or reflective functioning), thereby reducing vulnerability to maladaptive personality functioning. We therefore aimed to evaluate the strength of relationship between attachment insecurity and BPTs; in addition, we aimed to test the hypothesis that reflective functioning explains significant variance in the association between insecure attachment (avoidant and ambivalent) and BPTs in early adolescence. Focusing on attachment is crucial since although BPTs become more readily observable during adolescence, prospective findings suggest that relevant vulnerabilities, including early adversity and low maternal support may be detectable from the preschool years (11).

## 2 Method

### 2.1 Participants

All adolescents aged 11–14 years who requested a psychiatric assessment at a single outpatient Community Child and Adolescent Mental Health Service in Ioannina, Greece between June 2022 and September 2024 were invited to participate in the study. The psychiatric assessment was undertaken as part of routine clinical care and would have been conducted irrespective of participation in the study. Exclusion criteria were inability to read and write in Greek and presence of psychotic symptoms and moderate and lower levels of intellectual developmental disability.

The sample was composed of 112 early adolescents aged 11–14 years (mean = 12.8, SD = 1.16). There were 63 girls (56.3%) and 49 boys (43.7%). Adolescents were assessed by an experienced child and adolescent psychiatrist (with 20 years of clinical experience), and they were requested to complete study questionnaires. To minimize potential bias, the child psychiatrist who conducted the clinical assessment was blinded to the questionnaire scores. No missing data was identified. All parents and adolescents were informed about the purpose of the study, and they gave consent in written and verbally respectively. Ethical approval was granted by the Scientific Research Committee of the University General Hospital of Ioannina [approval number 2/28-01-2021]. This cross-sectional study was reported in accordance with the STROBE (Strengthening the Reporting of Observational Studies in Epidemiology) guidelines for observational studies.

### 2.2 Instruments

Borderline Personality Features Scale – Children (BPFS-C): The unidimensional modified 9-item Greek version of the BPFS-C was used (12). Adolescents scoring above the validated (12) Greek cut-off score of 26, were classified as “having high BPTs”. Internal consistency was good in the present sample (McDonald's *ω* = 0.81).

Attachment Questionnaire for Children (AQC): The AQC includes three brief descriptions that reflect children's feelings and perceptions about their relationships with other children. Children and adolescents are asked to read these descriptions and select the one that best describes them. Based on their choice, they are classified as having a secure, avoidant, or ambivalent attachment style (13).

Reflective Functioning Questionnaire (RFQ-8): The RFQ-8 was developed as a brief, easy-to-administer screening measure of reflective functioning (14). Although developed for adults, the tool has been used in adolescents as well (15, 16). Given the double scoring of some items, its factor structure is still open to debate (17). We performed a confirmatory factor analysis (data not shown) where the original two factor structure failed to fit our data. However, a unidimensional solution, based on the German version (18), dropping two items (Items 4 and 7) showed an excellent fit (RMSEA = 0.000, CFI = 1.000, TLI = 1.014) with an acceptable reliability (McDonalds *ω* = 0.74), considering the sample size of our study. This unidimensional scale could capture a continuum of uncertainty about mental states (high values) to absence of uncertainty (low values).

### 2.3 Statistical analysis

Categorical variables were summarized using percentages. The first analysis addressed the study aim of examining the association between attachment style and BPT group. The association was examined using a chi-square test of independence. *Post-hoc* pairwise comparisons of proportions with Bonferroni correction were conducted to further explore group differences. To test our hypothesis and exploring the associations among attachment style (independent variable), reflective functioning (mediator), and BPT classification (dependent variable) two separate mediation models were estimated. The first model evaluated the indirect effect of ambivalent (vs. secure) attachment, while the second model evaluated the indirect effect of avoidant (vs. secure) attachment, both models on BPT classification through reflective functioning. Indirect effects were estimated using nonparametric bootstrapping with 95% confidence intervals.

Analysis was performed using the software RStudio version 2024.12.0. Database sheet and the R code are available upon reasonable request.

## 3 Results

**Figure 1:**
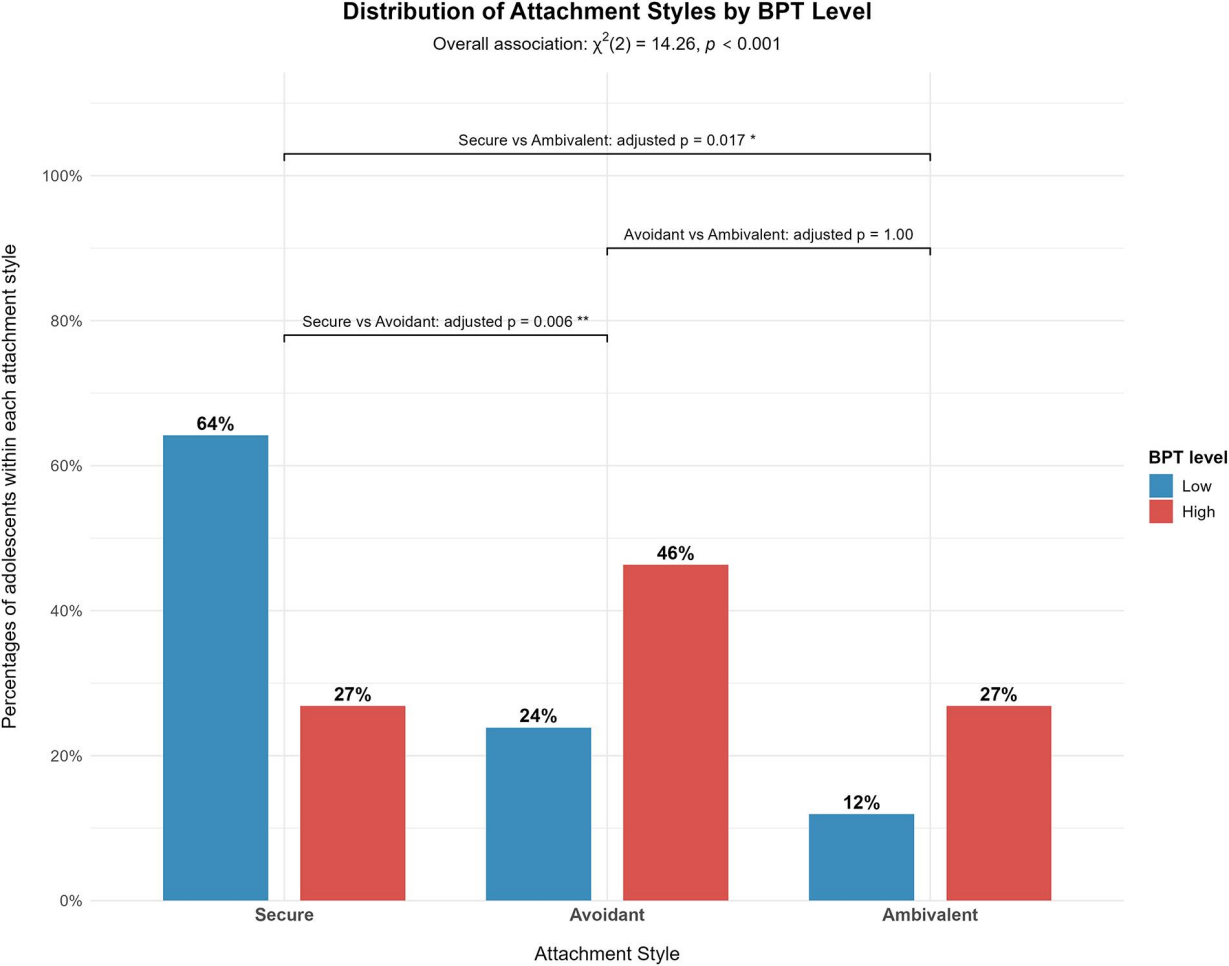
Distribution of attachment styles among adolescents with low and high borderline personality traits (BPTs). Percentages represent the distribution of attachment styles within each BPT group. Brackets show Bonferroni-adjusted pairwise comparisons. *p < .05; ** *p* < .01.

Our results showed that within the secure attachment group, 20.4% of adolescents were classified as having high BPTs, whereas 79.6% were classified as having low BPTs. Within the avoidant attachment group, 54.3% presented high BPTs, compared to 45.7% with low BPTs. Similarly, within the ambivalent attachment group, 57.9% were classified as having high BPTs, while 42.1% were classified as having low BPT levels. Additionally (Figure 1), among adolescents classified as having high BPTs, the distribution of attachment styles showed that 46% were characterized by an avoidant attachment, while 27% were classified as ambivalent and 27% as secure. As expected, on the other hand, among adolescents classified as having low BPTs, the distribution of attachment styles was markedly different from that observed in the high BPT group. Most participants with low BPTs exhibited a secure attachment style (64%), followed by an avoidant attachment style (24%), while a smaller proportion were classified as ambivalent (12%).

A chi-square test of independence indicated a significant association between attachment style and BPT group (*χ*^2^ = 14.26, *p* < 0.001). *Post-hoc* pairwise comparisons of proportions with Bonferroni correction showed that the secure attachment group differed significantly from both the ambivalent (*p* = 0.017) and avoidant attachment groups (*p* = 0.006), whereas no significant difference was observed between the ambivalent and avoidant groups (*p* = 1.00).

**Figure 2:**
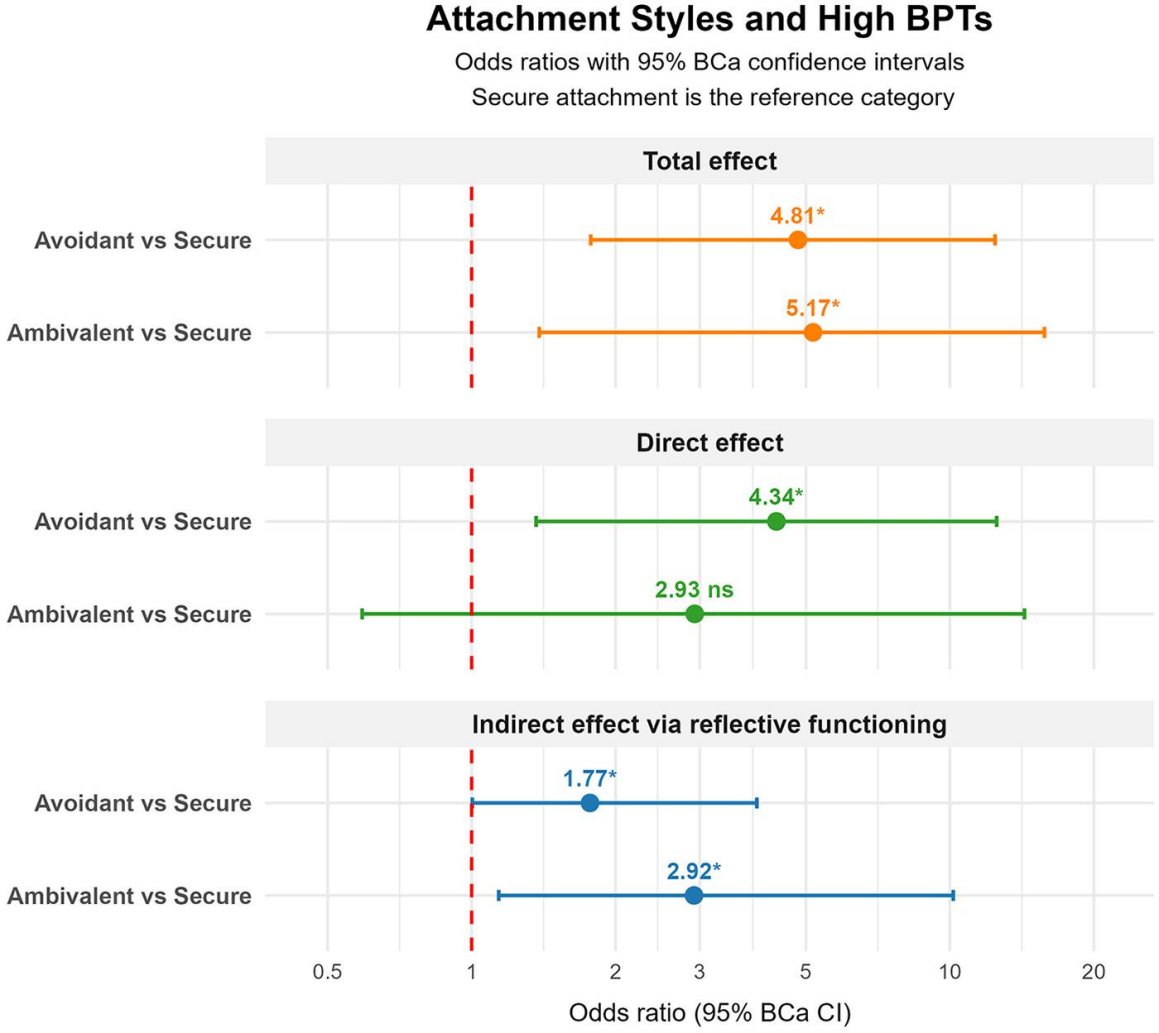
Direct, indirect, and total associations of attachment styles with high borderline personality traits (BPTs). The indirect effect represents the association through reflective functioning. The dashed line indicates OR = 1.00. * 95% BCa CI excludes 1.00; ns, not statistically significant.

A mediation analysis was conducted to explore whether reflective functioning accounted for significant variance in the association between attachment style and high BPTs, using secure attachment as the reference category (Figure 2). Regarding indirect effects, reflective functioning explained a significant amount of variance in the association between ambivalent attachment and high borderline personality traits (BPTs) [OR = 2.92, 95% BCa CI (1.14, 10.17)], indicating an increased likelihood of high BPTs through impairments in reflective functioning. In contrast, the indirect effect for avoidant attachment was smaller and reach only marginal statistical significance [OR = 1.77, 95% BCa CI (1.003, 3.95)]. With respect to direct effects, avoidant attachment showed a significant direct association with high BPTs [OR = 4.34, 95% BCa CI (1.36, 12.53)]. In contrast, the direct effect of ambivalent attachment was not statistically significant [OR = 2.93, 95% BCa CI (0.59, 14.32)]. Finally, total effects indicated that both insecure attachment styles were significantly associated with increased odds of high BPTs. Adolescents with an ambivalent attachment had more than five times higher odds of high BPTs compared to securely attached adolescents [OR = 5.17, 95% BCa CI (1.38, 15.77)], while those with an avoidant attachment showed a similarly elevated risk [OR = 4.81, 95% BCa CI (1.77, 12.43)].

## 4 Discussion

The present study investigated the relationship between attachment styles and BPTs in early adolescence, exploring the mediating role of reflective functioning. The findings provide evidence that insecure attachment styles are strongly associated with elevated levels of BPTs, while secure attachment appears to have a protective effect, mirroring results in adult populations (19, 20).

Adolescents with secure attachment were predominantly classified within the low BPT group, whereas high BPTs were markedly overrepresented among those with insecure attachment styles. This is consistent with the literature where insecure attachment is associated with elevated BPTs in adolescents BPTs (21). Caregiving characterized by emotional unresponsiveness and neglect-core features associated with insecure attachment-has been proposed as a significant developmental pathway to borderline personality pathology. Such relational environments may undermine the child's emerging capacity for reflecting other's mental states (10), and the downstream development of affect regulation and the development of a coherent sense of identity. In contrast, secure attachment relationships may reduce the vulnerability to BPTs (5, 22).

Mediation analysis offers deeper insight into the association of attachment insecurity to BPTs. Reflective functioning was shown to explain significant variance in the association between ambivalent attachment and high BPTs. While this study is cross-sectional, it tentatively suggests that, for adolescents with ambivalent attachment, impairments in the capacity to reflect other's mental states may represent a significant pathway through which attachment insecurity translates into BPTs. Ambivalent attachment is characterized by hyperactivating relational strategies, whereby the individual persistently seeks closeness, reassurance, and confirmation of availability. These behaviors are often accompanied by heightened emotional arousal (23). Within this emotional arousal, reflective functioning might lack the coherence and depth necessary for understanding the complex emotional states of self and others. Therefore, this impairment in reflective functioning might represent the pathway for the emergence of BPTs.

In contrast, avoidant attachment displayed a partially different pattern. Although a small indirect effect through reflective functioning was marginally significant, the direct association remained substantially stronger. This might be explained by the fact that avoidant attachment is characterized by efforts to maintain emotional distance and a strong preference for autonomy and self-reliance (24). Within this relational pattern, adolescents may downplay the importance of interpersonal experiences. As a result, they may appear to rely less on reflective functioning in close relationships, not because reflective functioning is impaired, but because relational disengagement reduces the perceived need to reflect on their own and others' mental states. Taken together, the differential mediation patterns observed across attachment styles underscore the heterogeneity of developmental pathways leading to BPTs in adolescence which may reflect the heterogeneity of the disorder. Furthermore, the marginally significant indirect effect of reflective functioning in the association between avoidant attachment and BPT should be further explored in future studies with large samples.

The association between ambivalent attachment and borderline pathology observed in the present study is consistent with findings from the adult literature (25, 26), where ambivalent attachment has been repeatedly identified as a correlate of borderline personality features, both directly and through impairments in reflective functioning. The picture is less straightforward for avoidant attachment. The noteworthy finding of the present study is the strong direct association between avoidant attachment and BPTs. This suggests that avoidant attachment constitutes a meaningful and relatively autonomous risk factor for borderline pathology in early adolescence, operating through mechanisms that are not primarily captured by reflective capacity. In this regard, our findings are only partially consistent with the available—and notably sparse for the avoidant attachment—evidence. In a recent study with a large sample (5), authors found a significant mediation through reflective functioning for both attachment anxiety and attachment avoidance in relation to borderline personality symptoms. The more attenuated indirect pathway observed in our study for avoidant attachment may reflect the earlier developmental stage of the present sample.

Limitations to this study include the small sample size that limits generalizability, its cross-sectional nature that does not allow for causal inferences and the classification of attachment by one self-reported question. The generalizability of the findings is further limited by the single-site, single-country design of the study. Furthermore, we did not account for several potentially important confounding factors known to be associated with borderline personality traits, including psychiatric comorbidity, childhood trauma exposure, and family rearing characteristics. Given the high prevalence of comorbidity and adverse childhood experiences among youth with borderline features, the observed effects may be partially influenced by residual confounding. Future studies employing large-scale, multicenter, and cross-ethnic designs are warranted to verify the reproducibility of these findings and establish their generalizability across diverse cultural and demographic contexts. The help-seeking nature of the sample should be considered when interpreting the findings. Participants had requested a psychiatric assessment and may therefore have had greater psychological distress, comorbidity, and interpersonal or family difficulties than adolescents in the general population. Accordingly, the observed levels of BPTs, attachment insecurity, and reflective functioning difficulties cannot be generalized to non-help-seeking adolescents. The magnitude of the associations may also differ in community samples, and replication across both clinical and nonclinical populations is needed. Nevertheless, the present study provides valuable insight into a developmental period during which preventive and early intervention efforts may be particularly important, not only for reducing the risk of emerging borderline personality disorder but also for mitigating the interpersonal and psychosocial consequences associated with borderline personality traits, especially within friendships and close relationships.

In conclusion, our study showed that insecure attachment styles are associated with elevated BPTs in early adolescence. Reflective functioning emerges as a potential mediator in this association, particularly for adolescents with ambivalent attachment, whereas its role appears more limited in the case of avoidant attachment. Clinically, our findings highlight the value of evaluating attachment and reflective functioning in early adolescents presenting borderline features (27). Moreover, they underscore the importance of attachment-informed and mentalization-based approaches tailored to different attachment styles. For adolescents with avoidant attachment, interventions may need to place greater emphasis on facilitating emotional engagement alongside strengthening reflective functioning, given that factors beyond reflective functioning appear to contribute substantially to the association with borderline features. In this context, interventions may need to be adapted to a more personalized one. Importantly, the findings support that early adolescence represents an important age group for prevention and early intervention. Strengthening reflective functioning and addressing attachment-related vulnerabilities during this developmental period may alter the trajectories of borderline personality pathology later in adulthood.

## Data Availability

The raw data supporting the conclusions of this article will be made available by the authors, without undue reservation.
